# Amplified Light Absorption with Nanomaterials for Enhanced Photoacoustic Imaging in Biomedical Research: A Review

**DOI:** 10.3390/bioengineering13040404

**Published:** 2026-03-31

**Authors:** Yong Duk Kim, Jijoe Samuel Prabagar, Dong-Kwon Lim

**Affiliations:** 1 KU-KIST Graduate School of Converging Science and Technology, Korea University, 145 Anam-ro, Seongbuk-gu, Seoul 02841, Republic of Korea; selapin@korea.ac.kr (Y.D.K.); joesam@korea.ac.kr (J.S.P.); 2 Department of Integrative Energy Engineering, College of Engineering, Korea University, 145 Anam-ro, Seongbuk-gu, Seoul 02841, Republic of Korea; 3 Brain Science Institute, Korea Institute of Science and Technology (KIST), Hwarang-ro 14-gil, Seongbuk-gu, Seoul 02792, Republic of Korea

**Keywords:** photoacoustic imaging, nanomaterials, biomedicine, imaging diagnosis

## Abstract

Recently, photoacoustic (PA) imaging has made a significant impact on biomedical imaging, providing detailed information on tissue structure and function by integrating optical and acoustic techniques. PA imaging can provide functional information at the cellular (e.g., oxygen saturation, hemoglobin concentration, metabolic rate) and molecular levels, owing to its substantial advantages over conventional imaging techniques. PA imaging is particularly useful for neuroimaging, cancer detection, and cardiovascular studies. Over the last decade, there has been a tremendous amount of research and development dedicated to nanomaterials that are ideal for PA imaging. Examples of nanomaterials include carbon-based and gold nanorods, both of which demonstrate greatly enhanced light absorption capabilities in the near-infrared range. Therefore, the properties of these materials make them perfect for achieving deep penetration into tissues. In addition, they exhibit biocompatibility, tunable optical properties, and enhance the acoustic signal for PA imaging, resulting in greater accuracy and precision in PA results. Researchers working in this area have focused on developing nanomaterials with fabrication capabilities, enabling real-time visualization of therapeutic events and enhancing light absorption. This review critically examines recent advances in nanomaterials for PA imaging, emphasizing strategies for signal enhancement and evaluating their impact on imaging performance, including imaging depth, photostability, and signal intensity, as well as their suitability for biomedical applications. Furthermore, complementary approaches for PA signal enhancement are discussed to provide a broader perspective and guide the selection and design of effective contrast agents for clinical and preclinical use.

## 1. Introduction

In the field of biomedical sciences, various noninvasive imaging techniques are available, which include positron emission tomography (PET), magnetic resonance imaging (MRI), ultrasound (US), and computed tomography (CT) [1,2,3,4]. Among them, optical imaging is particularly advantageous for observing and monitoring biological processes in organisms in real time. Since it does not use ionizing radiation, it is particularly appealing for biological research and medical applications [5,6]. The integration of multiple physical principles to improve image resolution, depth perception, acquisition speed, and three-dimensional reconstruction, thereby surpassing the capabilities of conventional instruments, has led to the development of hybrid imaging methods such as PET-coupled X-ray excited optical luminescence (XEOL) [7]. While optical imaging has significantly advanced, conventional methods remain inadequate for achieving improved visualization at the centimeter depth scale. The problem has been effectively addressed by the development of photoacoustic (PA) imaging, which combines optical contrast with ultrasonic resolution [8,9].

In biomedicine, PA imaging serves as an innovative technique that combines conventional optical imaging coupled with the high temporal and spatial resolution of US imaging, providing an integrated strategy for improved imaging [10,11]. This technique takes advantage of the PA effect, first described by Alexander Graham Bell [12]. In brief, when biological tissue absorbs light energy, it undergoes rapid thermoelastic expansion, generating ultrasonic waves [13]. A US transducer is operated to subsequently capture these waves and convert them into electric signals, facilitating the production of PA images. PA imaging works based on the light-absorption properties of the desired tissue, enabling noninvasive imaging. Given that PA signals are controlled by the spatial distribution of light absorption within the tissues, PA imaging shares the high contrast and sensitivity to tissue-specific properties with traditional optical imaging.

Usually, the contrast agents for PA imaging are characterized into endogenous chromophores and exogenous agents [14]. Endogenous chromophores, which include oxyhemoglobin, melanin, lipids, cytochromes, deoxyhemoglobin, collagens, and water, can absorb light naturally and are also capable of visualizing physicochemical changes [8,14]. However, their effectiveness is largely limited to optically transparent organs and tissues, and hemoglobin normally influences the PA signal, lowering specificity [15]. To enhance imaging contrast, specific contrast agents, particularly exogenous particles, have been researched, which has promoted the desire for high-performance PA materials. More sophisticated PA contrast agents, particularly nanomaterials that have better optical absorption properties through plasmonic resonance effects and other advantageous phenomena, have been made possible by recent advancements in nanofabrication techniques [16,17,18,19,20]. Therefore, to increase both the resolution and sensitivity of PA imaging, considerable effort has been made to develop contrast-enhancement techniques that incorporate a variety of exogenous agents, particularly nanomaterials.

Although earlier research has been included to provide essential contextual information, most of the literature examined in this review dates to the past decade. The Web of Science, Scopus, and Google Scholar databases were employed to perform an extensive literature search. The terms “photoacoustic imaging,” “nanomaterials,” “plasmonic nanoparticles,” “light absorption enhancement,” and “photoacoustic contrast agents” were used. Only English-language, peer-reviewed journal articles were considered.

Using a principles-to-application approach, this review provides readers with the foundational knowledge needed to understand subsequent advances by outlining the fundamental principles and key application areas of PA imaging. Research on nanomaterials as optical absorbers is advancing rapidly. This article presents the most important findings from recent research studies and reviews the ongoing advances in nanomaterials. In recent years, several review articles have addressed the fundamental concepts underlying PA imaging and contrast agents. Most of the reviews currently available concern contrast agents, imaging equipment, or certain types of materials. Therefore, the key objective of this review is to provide guidance and insight on which PA contrast agents to use, based on their ability to enhance light absorption and their performance. Although the primary emphasis is on nanomaterial-based strategies for enhancing light absorption, complementary approaches, including advancements in acoustic detection and signal processing techniques, are also discussed to provide a more comprehensive perspective on PA signal enhancement.

## 2. Fundamentals of PA Imaging

A PA signal is produced upon irradiation of biological tissues with short-pulsed laser light, during which chromophores within the tissue absorb the incident optical energy [21]. The absorbed energy induces a rapid localized temperature increase and thermal expansion, which in turn generates PA waves within the tissue [22,23]. These US waves are subsequently detected by ultrasound transducers and, after signal processing and image reconstruction, are visualized as two- or three-dimensional images.

PA imaging offers superior spatial resolution compared to conventional ultrasound, which usually has several millimeters of spatial resolution, whereas PA imaging can achieve resolutions down to several micrometers. This greater spatial resolution enables PA imaging to bridge the gap between optical imaging, which has a high contrast, and US, which provides deep tissue penetration [24,25]. The contrast in PA imaging is based on the wavelength of the light used to excite the tissue to be imaged. Because the optical absorption coefficient of the biological tissues depends on the molecular composition, each tissue exhibits a characteristic peak wavelength of absorption. By selecting an excitation wavelength that matches the absorption characteristics of the target tissue, the PA signal produced by that tissue is amplified, making it easier to see in relation to other tissues in the area. Light sources that emit monochromatic light in the ultraviolet, visible, and near-infrared (NIR) spectra are used to produce the excitation wavelengths required for PA imaging. These excitation wavelengths will vary between endogenous chromophores and exogenous contrast agents, allowing for a selective enhancement of the PA signals [26,27].

Biomolecules that are capable of absorbing visible light and generating PA signals from tissues are known as endogenous chromophores (e.g., DNA, RNA). Many types of biomolecules can act as endogenous chromophores, including nucleic acids, which have strong absorption peaks at approximately 260 nm [28]. The residues of aromatic amino acids that comprise many proteins (tryptophan, tyrosine, and phenylalanine) exhibit a strong absorption peak within the range of 250–300 nm [29], thereby permitting nuclei and cytoplasmic structures within tissues to be visualized using label-free methods with UV excitation. Hemoglobin is a chromophore within the visible to NIR-I spectral region, allowing assessment of vascular architecture, oxygen saturation, and processes associated with angiogenesis [30]. In the NIR-II spectral range, collagen and lipids are primary endogenous molecular targets that can be used to evaluate arterial disease and lipid-laden plaques. Melanin is also a robust endogenous chromophore in the NIR region; it absorbs light within the entire visible and near-infrared spectrum. Many applications in PA imaging have used melanin as a chromophore due to its broad absorption characteristics. Exogenous contrast agents are compounds that are administered to the body and used to increase the PA signal arising from physiological processes or to target particular physiological processes that can be monitored using PA signals [2]. Indocyanine green (ICG) and methylene blue have been approved by the U.S. Food and Drug Administration (FDA) for clinical use and are applied for tracking biodistribution in the gastrointestinal, lymphatic, and hepatobiliary systems [31]. Brilliant Blue G (BBG), an FDA-approved ophthalmic surgical dye, exhibits strong absorption in the visible spectrum; however, its limited absorption in the NIR region restricts its utility in deep-tissue imaging [32]. At the research stage, a variety of exogenous probes are under development, including metallic nanomaterials (e.g., gold nanorods, nanoshells, and nanocages) [33,34], carbon-based nanostructures (e.g., carbon nanotubes, graphene oxide, and carbon dots) [35], organic small molecules (e.g., cyanine dyes and boron-dipyrromethene (BODIPY) derivatives) [36], and semiconducting polymer nanoparticles [37]. The signal-to-noise ratio (SNR) and image contrast are enhanced by these agents while extending the scope of practical PA imaging.

## 3. Nanomaterials for Enhanced Light Absorption

### 3.1. Metallic Nanomaterials

#### 3.1.1. Gold Nanoparticles

Gold nanoparticles (AuNPs) are among the most promising materials, exhibiting strong localized surface plasmon resonance (LSPR) in the visible-light region [38]. AuNPs are widely utilized as exogenous PA contrast agents due to their numerous advantages, including tunable optical properties depending on size and shape, particle stability, relatively high biocompatibility, and facile surface functionalization [39,40]. As the aspect ratio of AuNPs increases, their optical absorption peak shifts toward longer wavelengths. Accordingly, anisotropic nanostructures with high aspect ratios, such as gold nanorods (AuNRs) or nanoprism-shaped particles, are frequently utilized [33,41,42]. However, exposure to high-intensity laser pulses employed in PA imaging induces photodamage to these particles [43,44], leading to structural alterations that collapse their NIR absorption and consequently render them unsuitable for stable PA imaging.

**Figure 1:**
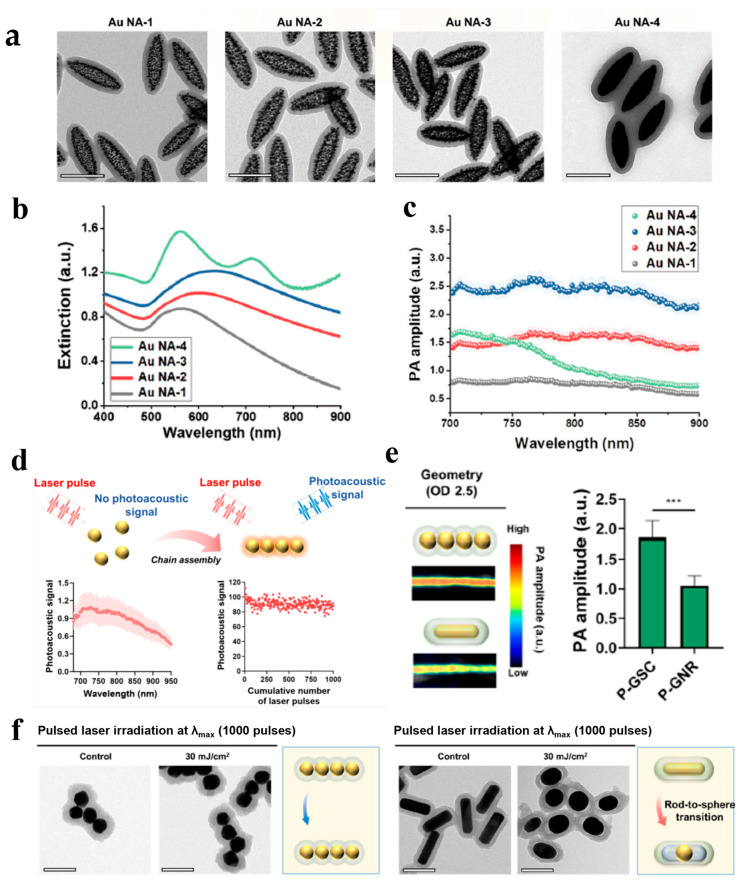
(**a**) TEM images of tunable Au NAs (scale bars 200 nm), (**b**) UV–vis–NIR spectra of Au NAs with tunable interparticle connectivity, (**c**) PA signals in the 700–900 nm spectral range from several Au NA solutions with the same Au mass concentration, ref. [45] Copyright 2023, Wiley. (**d**) Investigation of PA signal from polydopamine-coated (P)-GSCs, (**e**) Generation of PA signal from P-GSCs and P-GNRs at their peak absorption wavelength (OD 2.5, *n* = 5, *** *p* ≤ 0.001). (**f**) TEM images of P-GSCs and P-GNRs after pulsed laser illumination for 1000 pulses at a laser fluence of 30 mJ cm^−2^ (scale bars 100 nm), ref. [46] Copyright 2024, American Chemical Society.

To overcome the limitations of conventional AuNPs and AuNRs of PA imaging contrast agents, Kim et al. developed anisotropic Au nanoassembly (Au NA) by engineering particle connectivity based on Au nanospheres with diameters of approximately 6.8–10.2 nm [45]. Different Au NAs with differing diameters and interparticle separations were obtained (Figure 1a). UV–vis–NIR spectroscopy revealed that Au NA-3 exhibited expanded and red-shifted absorption peaks due to enhanced interparticle plasmon coupling, whereas fully connected Au NA-4 displayed shoulder peaks arising from its rod-like morphology. The results indicate the seed-mediated growth technique facilitates the fabrication of anisotropic Au NAs exhibiting adjustable interparticle connection, whereas their optical characteristics may be regulated by alternating plasmon coupling (Figure 1b). Specifically, assemblies exhibiting semi-connectivity produced 3.4-fold and 2.4-fold higher PA signals than those with no connectivity and full connectivity, respectively, and displayed strong absorption in the NIR (700–900 nm) region (Figure 1c). Among these, Au NA-3 with a semi-connectivity structure demonstrated robust structural stability and resistance to photodamage, keeping stable PA signal generation even at laser fluences exceeding the American National Standards Institute limits (ANSI) safety limit (31.7 mJ cm^−2^; tested up to 40 mJ cm^−2^). Additionally, Kim et al. addressed the limitations of conventional AuNP PA imaging by designing gold sphere chains (GSCs) through a one-step dopamine-mediated assembly of highly uniform, plasmonically coupled gold nanospheres (GNSs, 40 nm in diameter), followed by polydopamine encapsulation for enhanced stability [46] (Figure 1d). These GSCs, typically comprising four linked GNSs, exhibited over 80% higher PA signal and near-infrared (800 nm) absorption compared to AuNRs, and more than 550% higher PA signal than uncoated AuNRs (Figure 1e). Importantly, GSCs maintained consistent structural and optical integrity over 1000 consecutive imaging sessions under the ANSI safety limit (≤30 mJ/cm^2^) (Figure 1f), thus demonstrating superior photothermal durability compared to traditional PA agents, including nanorods and nanostars.

**Figure 2:**
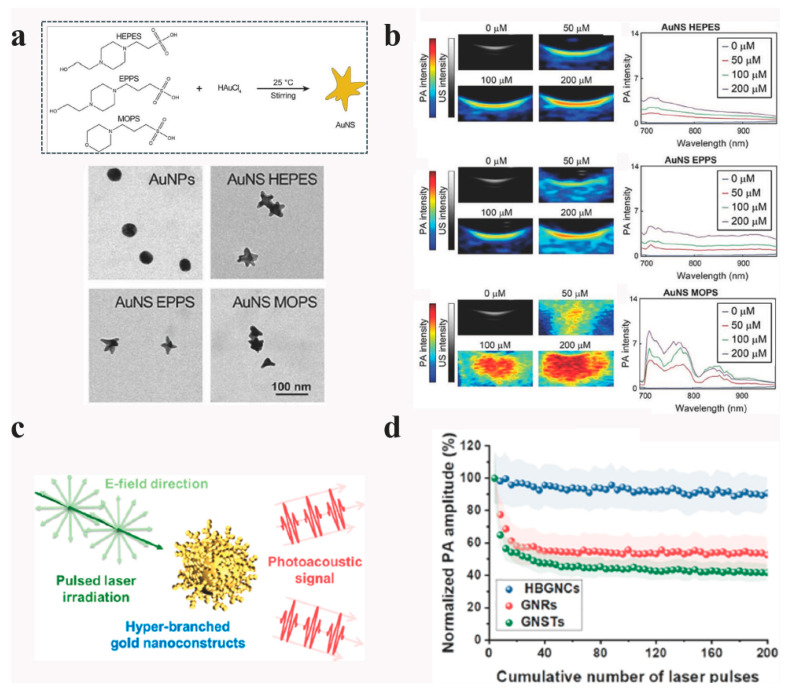
(**a**) Schematic representation of seedless growth of AuNS with Good’s buffers and TEM images of AuNPs and AuNS, (**b**) PA-US spectra and PA intensities at 710 nm of AuNS HEPES, AuNS EPPS, and AuNS MOPS from 0 to 200 μM, ref. [47] Copyright 2024, Springer Nature. (**c**) Illustration showing PA signal enhancement of hyper-branched gold nanoconstructs (HBGNCs), (**d**) PA signal generation from the different GNCs under pulsed laser illumination at a fluence of 10 mJ cm^−2^ (n = 4), ref. [48] Copyright 2023, American Chemical Society.

Zhang et al. developed biocompatible gold nanostars (AuNS) via a one-pot synthesis using Good’s buffers (HEPES, EPPS, and MOPS) (Figure 2a), systematically investigating the relationship between AuNS structure and PA imaging performance [47]. AuNS synthesized with MOPS buffer exhibited larger core sizes and more diverse branches compared to those made with HEPES or EPPS, resulting in 2–3 times higher PA signal in the 700–800 nm NIR region (Figure 2b), and up to 25 times higher signal than spherical AuNPs of similar size. Notably, melanin coating further enhanced the PA signal by up to 4.5-fold over PEG or chitosan coatings, attributed to melanin’s intrinsic photothermal properties. Excellent photostability is demonstrated by AuNS, preserving the PA signal after 10 min of constant irradiation, and exhibiting high biocompatibility in vitro and ex vivo, as supported by a persistent signal in biological medium. In a related study, hyper-branched gold nanoconstructs (HBGNCs, 35–75 nm) synthesized by seed-mediated growth and silver halide surface blocking achieved up to 90% absorption efficiency in the 700–900 nm NIR range and maintained strong PA signals across various light polarizations [48] (Figure 2c). HBGNCs produced 2.43-fold and 1.44-fold higher PA signals than AuNR and AuNS, respectively, and retained superior performance after PEGylation (Figure 2d). These constructs also demonstrated robust photostability, with no signal loss after 200 laser pulses (10 mJ/cm^2^), and maintained high surface area, durability, and shape stability, highlighting their promise as next-generation PA imaging probes for biomedical applications.

**Figure 3:**
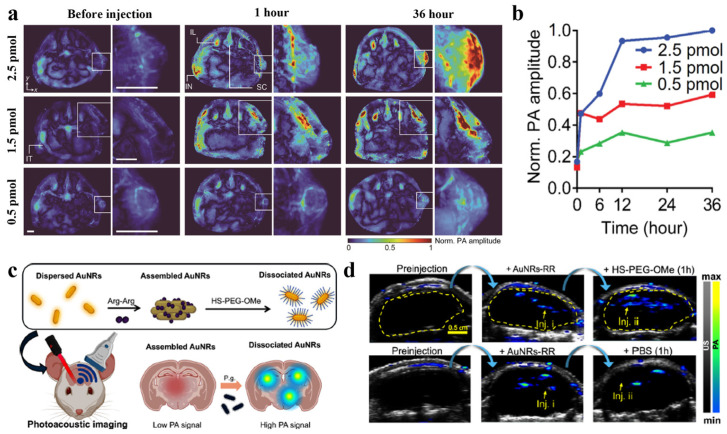
(**a**) Representative longitudinal PA maximum-amplitude projection (MAP) images of tumor slices over an elevational range of ~1 cm are shown for three different AC-GNS doses (0.5, 1.5, and 2.5 pmol), illustrating the corresponding in vivo tumor contrast (scale bar, 2 mm). (**b**) A plot depicts the temporal evolution of the mean PA signal within the tumor region following AC-GNS administration [49]. Copyright 2025, The American Association for the Advancement of Science. (**c**) A schematic summarizes the assembly and dissociation behavior of AuNRs and how this concept is exploited for PA imaging, including in vivo dissociation. (**d**) PA images acquired at 850 nm from the mouse brain show the in vivo dissociation. AuNRs–RR (20 μL, OD = 10) were first injected (Injet. i), followed by injection of HS-PEG-OMe (1.0 kDa, 20 μL, 1 mM) 5 min later (Injet. ii). Imaging performed 1 h after the second injection demonstrates dissociation in vivo (upper panel), whereas the lower panel shows a PBS-injected control for comparison [51]. Copyright 2025, American Chemical Society.

To further improve tissue penetration depth and SNR, considerable efforts have focused on red-shifting the absorption maxima of gold nanomaterials into the NIR, particularly the second NIR window (NIR-II). On this basis, high-sensitivity, high-precision PA imaging and PTT have been realized by engineering the shape and optical properties of gold nanostructures. In particular, silica-coated gold nanorods (SiO_2_-AuNRs) and armored core–gold nanostars (AC-GNSs) are tailored to exhibit strong plasmonic absorption in the NIR/NIR-II range, while thick silica or armored shells markedly enhance photothermal stability, thereby preserving both the localized surface plasmon resonance (LSPR) and PA signal under high laser fluence [49,50]. These design features enable real-time, quantitative mapping of temperature and thermal dose during deep-tissue PTT using PA readouts. Notably, SiO_2_-AuNRs tuned to 1064 nm maximize tissue penetration in the NIR-II region and maintain stable optoacoustic signals under both nanosecond-pulsed and continuous-wave (CW) irradiation with negligible photodegradation [50], whereas AC-GNSs support whole-body PA computed tomography (PACT) for simultaneous tracking of nanoparticle biodistribution and intratumoral temperature, achieving 100% survival in a bladder cancer model. Collectively, these studies demonstrate that amplifying light absorption in the NIR/NIR-II region, combined with improved photostability through structural and interfacial engineering of AuNPs, directly translates into enhanced in vivo PA image quality and therapeutic precision, suggesting a general design framework in which morphology control and robust coatings are exploited to maximize NIR absorption and sustain amplified PA signals over the course of treatment (Figure 3a,b) [49].

The activatable PA probe developed by Retout et al. exploits reversible changes in nanoparticle assembly rather than intrinsic absorption strength alone, focusing on dynamic modulation of the plasmon spectrum and PA output as a function of particle aggregation state [51]. Diarginine-induced assemblies of AuNRs exhibit plasmon coupling that alters their extinction spectrum and suppresses PA output, whereas protease-triggered cleavage of a PEG–peptide conjugate specific for arginine-gingipain (RgpB) induces dissociation of the clusters, restoring the LSPR of individual AuNRs and thereby enhancing the PA signal (Figure 3c,d). In vivo, this enzyme-responsive probe operates as an activatable contrast agent, providing up to approximately four-fold increases in PA signal intensity in the presence of active RgpB, while remaining largely silent in its absence. This strategy illustrates how dynamic assembly/disassembly of plasmonic nanostructures can be harnessed to achieve environmentally and enzymatically responsive on–off amplification of PA signals, underscoring the broad potential of stimulus-responsive nanomaterials for functional PA imaging in biomedical research. To highlight the role of Au nanostructures in enhancing PA signal generation. Table 1 summarizes representative Au-based nanomaterials, including nanorods, nanostars, and engineered assemblies, with emphasis on their optical absorption characteristics, photostability, and imaging performance.

#### 3.1.2. Copper Nanoparticles

Copper-based nanoparticles (CuNPs) have emerged as cost-effective and readily accessible alternatives to noble metal nanomaterials for antibacterial and sensing applications in the biomedical field [52]. In particular, owing to their excellent electrochemical properties and strong photothermal performance, CuNPs have been effectively utilized in a variety of cancer treatment strategies, including photothermal therapy (PTT) and photodynamic therapy (PDT) [53]. As light scattering in biological tissues increases, both optical intensity and the SNR of PA signals decrease. Consequently, a new trend has emerged toward enhancing PA imaging performance using contrast agents. Among the available contrast agents, CuNPs exhibit markedly enhanced PA signal amplification across diverse morphologies.

**Figure 4:**
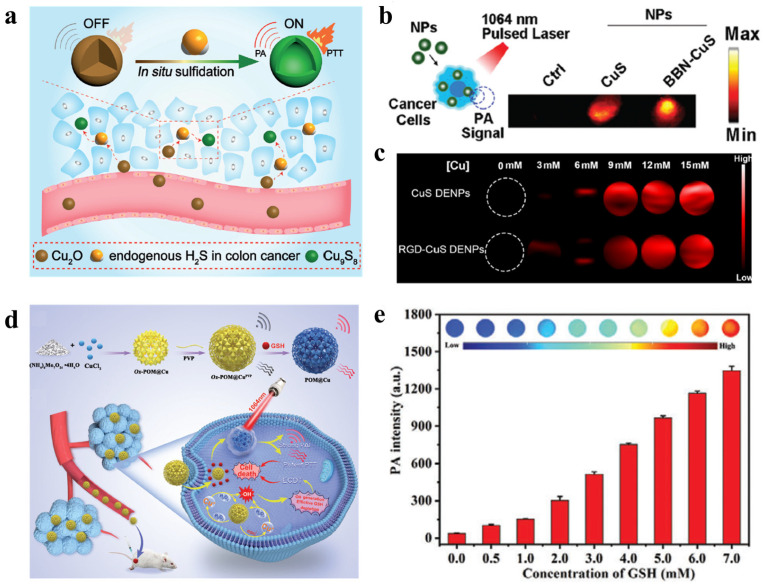
(**a**) Schematic illustration of endogenous H_2_S-triggered in situ transformation of Cu_2_O into Cu_9_S_8_ for PAI and PTT in colon cancer. Ref. [54] Copyright 2018, Wiley. (**b**) Representative PA images of C4-2 prostate cancer cells incubated with CuS nanoparticles (CuS NPs) or bombesin-conjugated CuS nanoparticles (BBN–CuS NPs), compared with untreated controls, ref. [55] Copyright 2021, Royal Society of Chemistry. (**c**) PAI images and corresponding signal intensities of RGD-functionalized CuS dendrimer-encapsulated nanoparticles (RGD–CuS DENPs) and non-targeted CuS DENPs at varying Cu concentrations [56]. Copyright 2021, American Chemical Society. (**d**) Schematic overview of the Ox-POM@Cu synthesis, PA imaging, and therapeutic mechanisms of the Ox-POM@Cu nanoenzyme. (**e**) PA signal intensities of Ox-POM@Cu after incubation with different concentrations of glutathione (GSH), acquired under laser excitation at λ = 1065 nm [57]. Copyright 2021, Wiley.

A recent study employs spherical Cu_2_O nanoparticles (~150 nm) that undergo sulfidation triggered by endogenous H_2_S present in tumor tissues, leading to their transformation into Cu_9_S_8_. This phase conversion generates a strong NIR absorption band, and the amplified absorption dramatically enhances the PA signal [54] (Figure 4a). In another research, copper sulfide (CuS) nanoparticles have been exploited to significantly enhance the sensitivity of PA by leveraging their strong absorption in the NIR-II window (~1064 nm). In a study by Hui-Chao Zhou et al., biomineralized ultrasmall CuS nanoparticles (average diameter 6–8 nm) were uniformly dispersed within an albumin matrix and exhibited intense NIR-II absorption at 1064 nm, resulting in a 6–8 fold amplification of the PA signal compared to water. This enhanced signal enabled precise visualization of the tumor, vasculature, and organ distribution [55] (Figure 4b). In another study, CuS clusters encapsulated within PAMAM G5 (poly(amidoamine) dendrimer, generation 5; featuring a core size of 2–3 nm and a hydrodynamic diameter of 30 nm) achieved a high photothermal conversion efficiency (η ≈ 49.8%) at 1064 nm, with the PA signal intensity increased by approximately sevenfold relative to untreated controls. This system was further applied as an innovative theragnostic platform for combining PA/thermal imaging and PTT/gene combination therapy in a TNBC (triple-negative breast cancer) tumor model in vivo [56] (Figure 4c). In both systems, the enhanced PA signals arise from a cascade mechanism associated with the high free-electron density of CuS. Strong localized plasmonic absorption in the NIR-II region leads to efficient heat generation, which induces thermoelastic expansion and subsequent emission of ultrasonic pressure waves responsible for PA signal amplification. Finally, a study was conducted using nanoparticles based on an oxidized molybdenum polyoxometalate (Ox-POM) framework doped with Cu ions. The resulting Ox-POM@Cu nanoparticles are spherical with an average diameter of approximately 25–30 nm. Cu doping of a Mo-based polyoxometalate induces the formation of oxygen vacancies, which increase the charge-carrier density and facilitate electron transport, thereby enhancing both redox activity and optical absorption intensity. Upon selective reaction with glutathione (GSH), which is abundant in the tumor microenvironment, a Mo^6+^→Mo^5+^ reduction occurs, leading to the generation and amplification of a strong NIR-II absorption band centered at ~1065 nm. Consequently, the PA signal intensity increases linearly with GSH concentration, a relationship that was experimentally validated [57] (Figure 4d,e). Table 2 provides a comparative overview of Cu-based nanomaterials, emphasizing their NIR absorption properties and potential as alternative plasmonic agents for PA imaging.

#### 3.1.3. Iron Oxide Nanoparticles

Iron oxide nanoparticles (IONPs) have been extensively investigated for a wide range of biomedical applications, including targeted drug delivery, MRI, and PTT [58,59]. However, IONPs without biocompatible surface coatings suffer from several limitations, such as excessive free radical generation, reduced ligand-binding efficiency, failure in effective drug delivery, and poor in vivo stability [60,61]. Therefore, these weak points can be effectively mitigated by introducing appropriate biocompatible coatings onto the nanoparticle surface.

Mandelis et al. reported that silica (SiO_2_) coating on Fe_3_O_4_-based IONPs simultaneously improved biocompatibility and PA contrast performance [62]. After silica coating, the particle size was uniformly controlled within the range of approximately 11 nm (8 nm Fe_3_O_4_ core, 3 nm SiO_2_ coating), and the nanoparticles remained stable without aggregation for over 24 h under serum and blood conditions. In PA measurements, the sensitivity of detection was further quantified, revealing minimum detectable concentrations of approximately 0.17 mg/mL at 5 mm and 0.23 mg/mL at 10 mm within the Intralipid matrix. Subsequently, studies converting the coating layer to mesoporous silica (mSiO_2_) have also been reported, in which mSiO_2_ was employed to enhance PA imaging performance and construct a multifunctional nanoplatform [63]. The mSiO-coating IONPs of spherical nanoparticles with an average diameter of approximately 260 nm, featuring mesopores of about 3.6 nm. The porous structure significantly enhanced photothermal accumulation effects. Under 532 nm excitation, the mSiO-coating IONPs dispersions generated strong PA signals that increased nonlinearly with nanoparticle concentration, reaching an average intensity of 0.0763 a.u. and an SNR of 28.76 dB at 17.6 mg/mL. At 1064 nm, the nanocomposite also produced a high-contrast PA image that clearly delineated the sample-containing tube while showing no detectable signal from the water-only control. Overall, surface modification of IONPs with silica-based coatings contributed to improved blood stability, suppression of nonspecific protein adsorption, and reduced organ toxicity. In addition, coating strategies using natural polymers such as rice starch or metallic layers such as gold have also been reported to further improve biocompatibility while amplifying PA signals, thereby broadening the biomedical applicability of iron oxide nanoparticles [64,65].

Additionally, Fe_3_O_4_-based magnetic nanoparticles (MNPs) were coated with natural rubber latex (NRL) to enhance NIR extinction and improve colloidal stability. At an identical concentration (0.50 wt%), the NRL-coated samples exhibited enhanced SNR in PA imaging. The SNR values of phantoms containing uncoated MNPs, NRL-100, and NRL-400 were 24.72 (0.51), 31.44 (0.44), and 33.81 (0.46) dB, respectively. These findings demonstrate that NRL coating significantly amplifies the PA signal by increasing optical absorption in the NIR region and enhancing the thermoelastic response [66]. Similarly, Periyathambi et al. reported that oleic acid-coated iron oxide nanoparticles (OA-IONPs, 12–14 nm) dispersed in an oil-based phantom exhibited concentration-dependent increases in PA amplitude, contrast, and SNR under LED-based PA images. At the highest tested concentration (1.5 mg/mL), the system achieved an SNR of approximately 11.3 dB in PA imaging. In this case, oleic acid coating enabled high-concentration, uniform dispersion in an organic medium, thereby enhancing effective optical absorption. As a result, these studies reveal a shared mechanism in which surface modification (e.g., silica, latex, or oleic acid coatings) enhances NIR absorption and dispersion stability, thereby yielding quantitatively higher PA contrast. Collectively, this strategy highlights surface-engineered IONPs as versatile platforms for PA signal amplification in biomedical imaging. To evaluate clinically relevant and biocompatible contrast agents, Table 3 summarizes IONPs, highlighting their multimodal imaging capabilities, stability, and translational potential.

### 3.2. Carbon-Based Nanomaterials

Carbon-based nanomaterials can be classified into two types, carbon nanotubes (CNTs) and graphene-based nanomaterials [68,69,70]. These materials have been widely studied as a capable contrast agent for PA imaging due to their NIR absorption. Though they have a molecular excitation coefficient lower than that of AuNPs, the ease of the fabrication and integration of carbon-based nanomaterials renders them intriguing for PA imaging applications. Further integrating the carbon-based nanomaterials with plasmonic metal nanomaterials can improve the PA intensity [9]. In research by Zerda et al., single-walled CNTs with cyclic Arg-Gly-Asp (cRGD) were developed and employed for tumor PA imaging [71]. Further coating single-walled CNT-cRGD with ICG dye enhanced the PA signal [72]. While maintaining high PA contrast and facilitating efficient molecular marker targeting in live animal models, this modification generated a 20-fold increase in optical absorbance at 780 nm compared with unmodified single-walled CNTs. In another study, Swierczewska et al. optimized hydrophobic single-walled CNTs for targeting tumors by linking a hyaluronic acid–5β-cholanic acid conjugate to folic acid. This modification significantly improved tumor uptake efficiency [73]. Later, PA imaging was employed to monitor the biodistribution and tumor accumulation of the modified single-walled CNTs (SWCNTs) in vivo.

**Figure 5:**
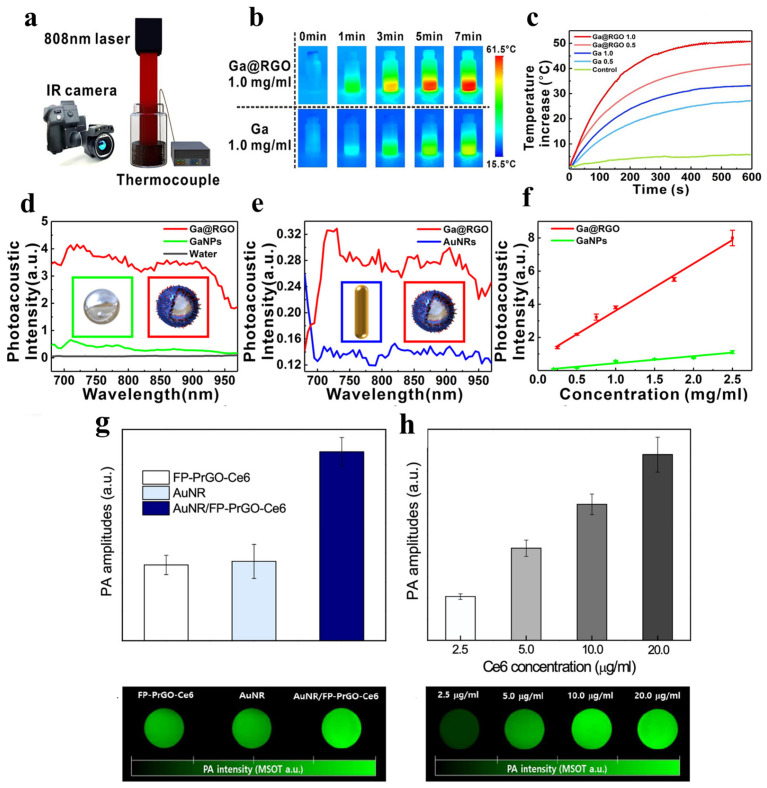
(**a**) Diagram of the photothermal experiment setup. (**b**) FLIR thermal imaging of Ga@RGO and Ga in ethanol (1.0 mg/mL) under laser exposure for 7 min at 1.5 W/cm^2^. (**c**) Temperature profiles of Ga@RGO and Ga in ethanol at varying concentrations (0.5 and 1.0 mg/mL) under 1.5 W/cm^2^ laser irradiation, with ethanol as the control. (**d**) PA signal intensity comparison between Ga@RGO and GaNPs at 1.0 mg/mL. (**e**) PA signal comparison of Ga@RGO and AuNRs at 0.1 mg/mL. (**f**) Correlation between PA intensity and concentration for Ga@RGO and GaNPs [76]. Copyright 2022, American Chemical Society. (**g**) PA response of FP-PrGO-Ce6, AuNR, and AuNR/FP-PrGO-Ce6 at equal concentrations, as evaluated by PA tomography (MOST), accompanied by corresponding PA images for each sample. (**h**) PA signal of AuNR/FP-PrGO-Ce6 across varying concentrations, along with its respective PA images [77]. Copyright 2021, American Chemical Society.

Further, graphene-derived nanomaterials constitute a prevalent category of carbon-based materials in PA imaging and have been rigorously studied in biomedical research owing to their unique physicochemical properties [74]. Graphene-based nanomaterials possess a larger surface area and are further versatile in a range of biological conditions than CNTs [9,75]. Zhang et al. facilitated the synthesis of core–shell nanostructures composed of liquid gallium coated with reduced graphene oxide (Ga@RGO) via a simpler one-pot sonication technique [76]. At 808 nm, their absorption cross-section reaches 457,884 nm^2^, significantly surpassing that of AuNRs (9656 nm^2^). Ga@RGO dispersions reached 50 °C within 5 min at a 1.0 mg/mL concentration when exposed to 808 nm laser radiation (Figure 5a–c). Notably, Ga@RGO nanocomposites exhibit exceptional light-harvesting properties throughout the NIR spectrum (680–970 nm) (Figure 5d,e). Composite nanomaterials made from reduced graphene oxide (rGO) and liquid gallium exhibit significant PA and photothermal properties (Figure 5f) because of the high photothermal conversion efficiency (42.4%) of rGO and its ability to absorb NIR light and the significant thermal expansion characteristics of liquid gallium when heated. By providing an excellent balance between optical absorption/thermal responsiveness, rGO’s ability to adjust the thickness of the rGO shell around the composite increases its durability against oxidative degradation and stability against particle agglomeration in biological environments. The work conducted by Lee and Kim illustrates the importance of rGO, especially its partially reduced form (PrGO), for enhancing the photothermal and PA capabilities of AuNR/FP-PrGO-Ce6 nanocarriers [77]. Upon exposure of PrGO to a laser at 880 nm, a significant photothermal response was observed due to the enhanced absorption of NIR light by PrGO compared to GO. The composite system reached 56.7 °C, indicating a synergistic interaction between PrGO and AuNRs, whereas PrGO alone reached 33.0 °C. With a photothermal conversion rate of 85.9%, this combined effect substantially exceeded the individual efficiency of PrGO (16.3%) and AuNRs (49.7%). Due to the increased NIR absorbance of PrGO and plasmonic resonance of AuNRs, the composite nanocarriers generated a signal intensity in PA imaging that was 2.4 times higher compared to that of bare AuNRs (Figure 5g,h).

Fullerene nanocrystals (FNCs) have emerged as promising materials for PA imaging due to their fundamental crystal structure and efficient light-to-heat conversion. However, their use in medicine has been limited because they do not disintegrate well in water and lack NIR absorption. This hybridization enhances NIR absorbance and enables energy transfer between Au and fullerene domains. This makes PA signals stronger and improves photothermal conversion, as in vivo studies showed strong imaging contrast, enabling clear tumor visualization [78]. Table 4 compares carbon-based nanomaterials, including graphene derivatives, focusing on their optical absorption, photothermal efficiency, and structural versatility.

### 3.3. Stimuli-Responsive Nanoparticles

A class of nanomaterials referred to as stimulus-responsive nanomaterials regulates their properties in response to certain internal or external stimulation. Stimulus-responsive nanomaterials can respond to external stimuli like radiation, magnetic forces, ultrasound, or electrical impulses as well as internal physiological changes within cells or tissues. Their incorporation into medical research has significantly enhanced several applications, especially in biomedical imaging. In a recent study, a multifunctional nanoplatform ADCuSi-FA consisting of ultrasmall CuS nanodots (1–2 nm) embedded within hollow mesoporous organosilica nanoparticles, which are further modified with folate-polyethylene glycol-silane to achieve both diagnostic and therapeutic functions, was designed [79]. Nanodots made from CuS are highly effective at absorbing light in the NIR II of the spectrum, which enables their use for PA imaging of deep tissues with both high resolution and sensitivity. When administered intravenously, the antibody-drug conjugate (ADCuSi-FA) accumulates at the site of a tumor, resulting in the generation of very strong PA signals that facilitate the accurate determination of tumor location and tracking treatment response. In addition to being effective for PA imaging, the same CuS nanodots can serve as photothermal agents by enhancing the PA contrast through NIR laser irradiation and inducing localized heating, leading to the release of copper ions and enhancement of the effect of chemodynamic therapy. The study conducted by Sivasubramanian et al. focused on engineering hybrid manganese oxide and mesoporous silica nanoparticles, coated with hyaluronic acid (HA), to respond to the overexpression of the enzyme hyaluronidase (HA-MnO@MSN) in many tumor types [80]. Degradation of the HA coating on the nanoparticles via an enzyme permits controlled access of water to the MnO core, enhancing T1 contrast for MRI and catalyzing the transformation of hydrogen peroxide within the tumor to oxygen as well. In addition, the continuous production of oxygen allows for alleviation of hypoxia and the formation of oxygen gas bubbles, which serve to improve the PA signal, thereby allowing real-time assessment of oxygen saturation in the tumor. The combination of tumor-targeting ligands, enzyme responsiveness, and catalytic oxygen modulation within these nanoparticles provides a dual-modality imaging platform, enabling structural imaging of the tumor with MRI and functional imaging of its oxygenation with PA imaging. To illustrate advanced strategies for activatable and targeted PA imaging, Table 5 summarizes stimuli-responsive nanomaterials, highlighting their responsiveness to environmental triggers and their ability to enhance imaging specificity.

### 3.4. Robustness of the PA Nanomaterials

While engineering nanomaterials with improved optical absorption has advanced significantly, the robustness and reliability of these systems remain a crucial factor for dependable PA performance. Signal reproducibility is directly affected by photostability under constant laser exposure, durability against structural modification, and stability in biological environments. While semiconducting and hybrid systems must preserve structural and optical properties over extended imaging, plasmonic nanostructures can encounter morphological alterations at high laser intensity, changing LSPR characteristics. Moreover, long-term colloidal stability and batch-to-batch consistency are essential for accurate quantitative PA measurements [85]. For instance, AuNRs have tunable plasmonic behavior and strong optical absorption; their photostability is frequently restricted by thawing or altering under strong radiation. Strategies like silica shell deposition, polymer encapsulation, and size reduction have been employed to address these effects [86]. These strategies greatly increase thermal resilience and reduce structural damage, supporting reliable and long-term PA functionality. To improve translational reliability, future studies should focus on standardized performance testing and systematic stability assessment.

Based on the presented studies, it is evident that different classes of nanomaterials offer distinct benefits varying depending on the PA imaging application. Plasmonic nanomaterials, particularly Au-based, reliably present the highest optical absorption and photothermal conversion efficiency, making them ideal for applications requiring strong signal amplification and high sensitivity. However, their limited biodegradability and potential long-term accumulation remain a key challenge for clinical translation. In contrast, organic and carbon-based nanomaterials offer improved biocompatibility and structural flexibility, although they may exhibit comparatively lower photostability or signal intensity. Additionally, nanomaterials operating in the NIR-II window facilitate deeper tissue penetration and higher permissible laser exposure, making them more suitable for deep-tissue imaging. Therefore, the selection of PA contrast agents should be guided by application-specific requirements, balancing signal intensity, imaging depth, and translational feasibility.

## 4. Signal Amplification

It is crucial to amplify the PA signal for better depth of imaging, sensitivity, and specificity. Various strategies can be employed to improve the amplification process, which include optical excitation, plasmonic/nanophotonic enhancement, acoustic detection improvements, and advanced signal processing. The following section provides a detailed overview of these enhancement strategies.

### 4.1. Optical Excitation Method

Exploiting the nonlinear effects—e.g., Grueneisen relaxation—by delivering sequential or multiple laser pulses can enhance the PA signals. Two compactly spaced pulses to thermally tag absorbers are employed in Grueneisen relaxation PA microscopy to increase the intensity of the PA signal and enhance axial resolution [87,88]. The signal amplitude can be further increased by long-laser-pulse-induced dual PA and quasi-continuous-wave via heat accumulation, resulting in better contrast. Another approach is using the longer wavelengths (NIR-II), which limits tissue scattering and enhances the maximum permissible exposure, resulting in better imaging and a higher intensity signal [89,90]. NIR agents such as phthalocyanines, semiconducting polymer nanoparticles, and nickel dithiolene dyes have demonstrated imaging depths beyond 5.0 cm and high SNR in preclinical models [91].

### 4.2. Plasmonic and Nanophotonic Enhancement

Plasmonic nanoparticles aggregation, such as AuNRs and nanospheres, can enhance the local electromagnetic fields and the generation of heat, resulting in an increase in PA signal intensity. Silica-coated AuNPs prevent unnecessary plasmon coupling and uphold photostability, whereas the PA signal increases remarkably with aggregation [19,92]. Furthermore, engineered nanostructures, which include GSCs, core–shell assemblies, and hyperbranched constructs, present strong amplification of the PA signal, tunable absorption, and enhanced photostability. In research, GSCs showed enhanced photostability and absorption efficiency compared to traditional nanorods, maintaining stable signals. In a study, the plasmonic property of Au nanoparticles was exploited in enhancing the light absorption for PA imaging [93]. The close interaction of the particles initiated plasmon coupling when the DNA aptamer promoted the AuNPs to aggregate amid matrix metalloproteinase-9 (MMP-9). Their absorption of light in the initial NIR-I window, a spectral band where biological tissues have comparatively low absorption, was altered and improved by this coupling effect. Consequently, more light energy was absorbed by the aggregated nanospheres and transformed into more intense acoustic signals. Both in vitro and in vivo, this amplification of the PA response made it possible to detect MMP-9 function in the tumor microenvironment with extreme sensitivity. In another study, titanium nitride@Au (TiN@Au) core–shell nanorods were employed to enhance the light absorption property using their plasmonic property [94]. NIR absorption efficiency is significantly improved by the LSPR produced by the coupling between the TiN core and the Au shell, which amplifies the local electromagnetic field. Stronger photothermal conversion because of this increased absorption results in stronger PA signals and more noticeable thermoelastic expansion. Additionally, the Au shell enhances field confinement and scattering, increasing the absorption cross-section while providing effective energy transfer. The nanorods are excellent contrast agents for PA imaging with superior signal intensity and deeper tissue penetration because they maximize absorption and thermal output by optimizing the shell thickness.

### 4.3. Acoustic Detection Improvement

Piezoelectric, capacitive micromachined, and piezoelectric micromachined ultrasound transducers produce high sensitivity, miniaturization, and broad bandwidth for portable devices [95,96]. Optical and acoustic axes can be co-aligned by transparent ultrasound transducers and resonators, resulting in better imaging and patient comfort [97]. Further, non-contact imaging is possible by PA remote sensing, which limits the risk of contamination and allows functional imaging as well as elastography. In a recent investigation, the acoustic detection in PA imaging was improved by integrating commercial piezoelectric transducer arrays and capacitive micromachined ultrasonic transducer (CMUT) arrays into a dual-modality PA tomography and ultrasound system [98]. The comparison showed that both arrays achieved similar spatial resolution in phantom tests. However, the CMUT array offered better sensitivity for detecting hemodynamic responses triggered by visual stimuli. It captured activity not just in cortical areas but also in deeper subcortical regions like the superior colliculus. This improvement indicates that CMUTs, with their lightweight silicon-based design and scalability, can match or even exceed traditional piezoelectric arrays in neuroimaging. Recent research by Tang et al. improved acoustic detection by replacing traditional ultrasound transducers with a non-contact PA remote sensing (PARS) method. The system used a small all-fiber probe with built-in optical components. It eliminated the need for coupling media like water or gel, reduced background noise, and improved sensitivity. This design allowed for stable, portable operation and enabled clear, high-resolution vascular imaging in vivo. It marked an important step forward in acoustic detection efficiency and practicality.

### 4.4. Signal and Image Processing

Pre- and post-processing techniques, which include wavelet transform, single value decomposition, averaging, and deconvolution, enhance the image resolution. However, it can be computationally demanding and can also result in the loss of signal [99,100]. Deep-learning techniques such as convolutional neural networks, learned spectral decoloring, and U-Net architectures show better denoising, quantitative accuracy, and artifact reduction in the estimation of functional tissue properties when compared to conventional linear unmixing [101]. Deep learning also enables multimodal data fusion, real-time reconstruction, and super-resolution. In a study, deep learning was utilized to improve the volumetric PA imaging by introducing a 3D fully dense U-net (3DFD U-net) altered for linear array-based tomography [102]. Compared to conventional reconstruction methods, the 3DFD U-net restored true vessel sizes, reduced electromagnetic interference noise and artifacts, improved contrast in deeper regions, and partially recovered features lost due to the limited-view problem. These improvements enabled the generation of clear, continuous 3D vascular images in human palms, arms, feet, and breasts, demonstrating significant potential for clinical applications such as biometric identification, foot ulcer evaluation, and breast cancer imaging. Although this review primarily emphasizes nanomaterial strategies for amplified optical absorption, recent developments in machine-learning-assisted data analysis may further enhance the interpretation and amplification of PA signals.

## 5. Clinical Translation, Safety, and Standardization Considerations

Despite substantial advances in enhancing light absorption with nanomaterials, clinical translation of PA contrast agents remains challenging. For inorganic nanomaterials containing heavy metals in particular, where prolonged biodistribution, clearance pathways, and possible accumulation must be carefully evaluated, toxicological evaluation is crucial [103]. However, semiconducting organic nanomaterials and FDA-approved dyes are generally considered more promising owing to their biodegradability and established safety profiles.

Further challenges in translation involve batch-to-batch consistency and synthesis reproducibility, since minor modifications to phase composition, surface chemistry, or size distribution can significantly alter optical absorption and photothermal efficiency [104]. Therefore, scalable production techniques and standardized characterization protocols are essential. Additionally, conditions for laser exposure must adhere to safety standards, such as the ANSI maximum permissible exposure (MPE) guidelines. Depending on wavelength and pulse duration, laser efficiency typically limits values to around or below ~20 mJ cm^−2^ in the NIR window, which is often employed for PA imaging [105]. In a recent study, ICG was employed as an organic dye for PA imaging [106]. Because it is FDA-approved and has promising properties such as biodegradability, biocompatibility, and photostability, ICG was used in the investigation. When conjugated with RGD peptides (ICG-RGD), it selectively targets choroidal neovascularization without causing systemic or ocular toxicity. In vitro assays showed minimal cytotoxicity even at high concentrations, while in vivo studies in rabbits demonstrated normal liver and kidney function, stable body weight, and no histological evidence of organ damage. Moreover, the laser energy applied during imaging was kept below ANSI safety limits, ensuring safe long-term visualization without photobleaching or tissue damage. Strong-signal-amplification nanomaterials that meet these safety requirements are especially appealing for medical applications.

Although most nanomaterial-based PA contrast agents are still in the preclinical stage, some nanomaterials have already been implemented in clinical settings, demonstrating the translational potential of nanomaterials in bioimaging. In addition to being widely used as contrast agents for MRI, superparamagnetic iron oxide nanoparticles (SPIONs), like ferumoxytol, are clinically approved for iron supplementation. They have also gained interest in using them as multimodal agents for PA imaging [62,107,108]. Similarly, liposomal nanocarriers, represented by clinically approved formulations such as liposomal doxorubicin (Doxil^®^), highlight clinical safety, scalability, and regulatory acceptance of nanoparticle-based delivery systems while offering versatile platforms for incorporating acoustic imaging agents [109]. Building on this clinically established platform, Wood et al. designed a liposome-encapsulated indocyanine green J-aggregate agent (“PAtrace”) composed exclusively of FDA-approved components and quantitatively demonstrated improved spectral unmixing performance at depth and targeted tumor accumulation, thereby illustrating the potential of a truly “clinically translatable” molecular PA contrast agent [110]. These clinically endorsed nanomaterials present important precedents for the safe use of nanomaterials in humans and may facilitate the future translation of nanomaterial-based PA contrast agents into routine clinical imaging applications.

## 6. Future Perspective and Conclusions

In recent years, a rapid development has been observed in the field of PA imaging through the utilization of nanomaterials. Specifically in biomedical imaging, amplified PA imaging is considered promising due to its ability to provide in-depth, enhanced, and molecular-level imaging of biological tissues. PA imaging can transform clinical diagnostics, therapy monitoring, and fundamental biomedical research through synergistic advances in contrast agents, signal amplification strategies, and image reconstruction. While significant challenges remain, especially in achieving optimal sensitivity, depth penetration, and clinical translation, recent advances in nanotechnology and PA imaging, combined with artificial intelligence, are rapidly closing these gaps. The PA imaging system should be an immediate, steady, and precise system for real-time imaging, data acquisition, and analysis, particularly for quantitative data analysis. The future of PA imaging will be influenced by ongoing interdisciplinary collaboration, safety and regulatory frameworks, and the translation of innovative technologies into robust, accessible medical tools.

Further, the effectiveness of optical absorption and PA signal production is influenced by the purity and phase integrity of nanomaterials, as well as their structural and compositional properties. Impurities such as unreacted precursors, structural defects, size distribution, or phase mixtures can affect optical absorption. Decreased reproducibility due to these impurities will limit the effectiveness of a material as a contrast agent. Therefore, to achieve consistent signal enhancement and to establish high-performance contrast agents for biomedical applications, it is critical to use controlled synthesis methods and complete physicochemical characterization for each nanomaterial produced. Future work that quantitatively evaluates the correlation between nanomaterial purity and the PA effect will help establish standardized benchmarks for developing high-performance contrast agents for medical use.

In summary, the increased use of PA imaging is accompanied by both great opportunities and challenges. It is expected that this technology will grow to be more actively involved in the multidisciplinary fields of advanced diagnostic instruments and functional materials as it moves closer to human health care.

## Figures and Tables

**Table 1:** Reports on AuNPs for PA imaging.

Imaging Contrast Agents Type	Size and Shape	Light Source	PA Signal	Stability	Target and Applications	Ref
Au nanosphere assemblies	Sphere (6.8–10.2 nm)	NIR (700–900 nm)	Semi-connected: 2.5× (vs. nanorods), 1.3× (vs. nanostars)	Stable for >1200 laser pulses (≤40 mJ/cm^2^, ANSI)	Strong and stable PA contrast agent design for longitudinal PA imaging at or above clinical laser fluence limits	[45]
Polydopamine-coated gold sphere chains	Sphere (40 nm)	NIR (800 nm)	>550% higher PA signal than uncoated AuNRs	Stable for >1000 laser pulses (≤30 mJ/cm^2^, ANSI)	High-contrast, photostable PA contrast agent for longitudinal molecular PA cancer imaging in vitro and in vivo	[46]
Gold nanostars	Nanostars (45 ± 5 nm)	NIR (700–800 nm)	MOPS-AuNS: 19–25× vs. spherical AuNP	Stable for 10 min continuous irradiation; 48 h in FBS	Melanin shells are used to maximize the PA signal in the 700–800 nm region for in vitro and ex vivo imaging	[47]
Hyper- branched gold nanoconstructs	branched gold nanoconstructs (35–75 nm), branch thickness: ~6 nm	NIR (700–900 nm)	PA signal: 2.43× vs. GNRs, 1.44× vs. GNSTs	Stable for 200 pulses (10 mJ/cm^2^)	Blackbody-like PA agent with polarization-independent strong NIR absorption, high PA output, and excellent photostability.	[48]
Silica- coated AuNRs	AuNRs (length of 45–50 nm and a diameter of 15–20 nm, an aspect ratio of roughly 2.5–3.0); silica shell about 15 nm thickness	NIR-II (1064 nm)	In deep tissue, PA intensity increases approximately linearly with temperature up to ~50 °C	No photodegradation at ns 9 mJ/cm^2^, structure maintained even after CW 16 W/cm^2^	Deep-tissue, real-time optoacoustic monitoring of photothermal therapy,	[50]
Armored core–gold nanostars	Diameter ~98 nm (range 106~125 nm across formulations), hollow/caged nanostar + outer shell (shell thickness tunable ~1–10 nm).	NIR (800 nm)	AC-GNS produces a strong PA contrast (In vivo PACT successfully tracked doses 0.5–2.5 pM.)	PA signal persists even after repeated PACT+ high-fluence PTT, with no long-term toxicity	Infection diagnosis and real-time evaluation of gingipain inhibitors for Alzheimer-related drug development.	[49]
Activatable AuNR assemblies	Diarginine-assembled AuNR clusters with PEG–peptide	NIR (850 nm)	Up to ~4-fold PA signal increase upon protease-triggered dissociation	Structurally stable under safe fluences; activation occurs specifically in the presence of the target protease	PA-guided precision photothermal therapy of solid tumors	[51]

**Table 2:** Comparison of CuNPs for PA imaging.

Imaging Contrast Agents Type	Size and Shape	Light Source	PA Signal	Stability	Target and Applications	Ref
H_2_S-activated Cu_2_O/CuS probe	Hollow nanoparticles (21–26 nm)	NIR (808 nm)	Strong “turn-on” PA signal after sulfidation; rapid and significant increase vs. Cu_2_O	Stable in physiological pH; selective activation by endogenous H_2_S	Colon cancer theragnostic: PA imaging-guided PTT	[54]
CuS nanoprobes	spherical (6–8 nm), hydrodynamic diameter increased to ~16.9 nm	NIR-II (1064 nm)	6–8× higher PA signal vs. water	High colloidal stability in serum; good biocompatibility	Tumor, vascular, and organ imaging using NIR-II PA imaging	[55]
RGD-CuS DENPs	Core: 4.2 nm (spherical), dendrimer-entrapped CuS NPs	NIR-II (1064 nm)	7× stronger PA signal vs. untreated control	Stable dendrimer-encapsulated structure in physiological media	TNBC tumor model: NIR-II PA imaging-guided PTT + gene therapy	[56]
Ox-POM@Cu Nanoenzyme	spherical (~5 nm)	NIR-II (1064 nm)	PA signal increases linearly with GSH; strong NIR-II activation	High hydrophilicity and stability; oxygen-vacancy-enhanced electron transfer	TME-activated PAI + synergistic CDT/PTT cancer therapy	[57]

**Table 3:** Comparison of IONPs for PA imaging.

Imaging Contrast Agents Type	Size and Shape	Light Source	PA Signal	Stability	Target and Applications	Ref
SiO_2_@Fe_3_O_4_ core–shell	Spherical (~11 nm)	NIR-II (1064 nm)	Detected 0.23 mg/mL at 10 mm within the Intralipid matrix	Stable in serum, no aggregation	Biocompatible PA contrast agent for deep tissue imaging and detection of silica-SPIONs in dense muscle (avian pectus, murine quadriceps)	[62]
mSiO_2_@Fe_3_O_4_ core–shell	spherical (~260 nm)	(532 nm and NIR-II (1064 nm))	Up to 0.0763 a.u., 28.76 dB SNR at 17.6 mg/mL at 532 nm)	long-term dispersion stability	Potential PA contrast agent providing good spatial and depth resolution for biomedical imaging	[63]
NRL-coated magnetic nanoparticles	Spherical, NRL-coated magnetic nanoparticles (15–20 nm)	NIR (750 nm)	At 0.50 wt%: contrast 28.81 dB (NRL-400)	Improved colloidal stability and magnetic response in aqueous phantoms	NPs for MMUS, PA imaging, and magnetic hyperthermia	[66]
Oleic acid- coated iron oxide nanoparticles	Spherical (OA-IONPs: 12–14 nm)	NIR LED arrays (850 nm)	At 1.5 mg/mL: PA SNR ≈ 11.3 dB; PA signal increases with concentration	Stable dispersion in oil-based phantom, suitable for repeated MMUS/PA measurements	Contrast agent for MMUS and PA imaging in non-aqueous/oil-based phantoms, with potential translation to specialized biomedical imaging setups	[67]

**Table 4:** PA imaging of carbon-based nanomaterials.

Imaging Contrast Agents Type	Size and Shape	Light Source	PA Signal	Stability	Target and Applications	Ref
Ga@RGO core–shell NPs	Liquid Ga core with RGO shell (~660 nm)	NIR (680–970 nm)	~5× higher PA signal than Ga NPs and ~2× higher than AuNRs; gain ~25 dB	Shell prevents coalescence; chemically stable	PA contrast agent and photothermal platform for cancer theranostics	[76]
AuNR/FP-PrGO-Ce6 nanocarriers	AuNRs on PrGO sheets with FA-PEG and Ce6 (~387 nm)	NIR (680–900 nm)	>2.4–2.5× higher PA amplitude than bare AuNR; increases with concentration	Stable in aqueous/serum media; stable over repeated laser on/off cycles	Targeted PA imaging and synergistic PDT/PTT of folate-receptor–positive tumors	[77]

**Table 5:** PA imaging of various simulative responsive nanoparticles.

Imaging Contrast Agents Type	Size and Shape	Light Source	PA Signal	Stability	Target and Applications	Ref
Cu^2+^-BSA-Gox@PANI (dual H_2_S/glucose–responsive)	Uniformly distributed spherical (~210 nm)	NIR II (1064 nm)	2× (vs bare PANI)	Stable for six 808 nm laser irradiation on/off cycle at 1.5 W/cm^2^	Precise diagnosis and photothermal-starvation synergistic therapy of colon cancer	[81]
AuNNPs–Ag_2_S vesicle (pH-responsive hybrid vesicle)	Assembled vesicle (~190 nm)	NIR-II (950–1700 nm)	9-fold increase compared to baseline after accumulation in tumors	Engineered for single use and not stable in an acidic microenvironment	Dual-mode NIR-II PA/fluorescence imaging–guided precise radiotherapy	[82]
AuNR@PNIPAM-VAA nanogel (low pH and thermal co-activated)	Nanorods with encapsulated sphere (~100 nm)	NIR II (1064 nm)	Shows ~1.6-fold enhancement with an increase in temperature in the simulated microenvironment	Stable for 6 laser irradiation on/off cycles at 0.75 W/cm^2^	Tumor-specific, switchable NIR-II PA imaging for low-pH tumor microenvironment detection and dynamic monitoring	[83]
AuNRs@TFF (GSH-responsive AuNRs with tannic-acid–Fe network)	Nanorods (~70-80 nm)	NIR II (1064 nm)	5-fold higher than bare AuNRs in vitro and ~3.5-fold higher in vivo	Stable for 5 consecutive laser irradiation on/off cycles at 1.0 W/cm^2^	NIR-II PA-guided synergistic chemodynamic therapy (CDT) and photothermal therapy (PTT) for tumors	[84]

## Data Availability

No new data were created.

## Abbreviations

The following abbreviations are used in this manuscript:

PA	Photoacoustic
PET	Positron emission tomography
MRI	Magnetic resonance imaging
US	Ultrasound
CT	Computed tomography
NIR	Near-infrared
ICG	Indocyanine green
BBG	Brilliant blue G
SNR	Signal-to-noise ratio
AuNPs	Gold nanoparticles
AuNRs	Gold nanorods
ANSI	American National Standards Institute limits
GSCs	Gold sphere chains
GNSs	Gold nanospheres
AuNSs	Gold nanostars
HBGNCs	Hyper-branched gold nanoconstructs
CuNPs	Copper-based nanoparticles
PTT	Photothermal therapy
PDT	Photodynamic therapy
CuS	Copper sulfide
Ox-POM	Oxidized molybdenum polyoxometalate
GSH	Glutathione
IONPs	Iron oxide nanoparticles
SiO_2_	Silica
mSiO_2_	Mesoporous silica
CNTs	Carbon nanotubes
Ga@RGO	Gallium coated with reduced graphene oxide
rGO	Reduced graphene oxide
